# Strengthening the governance of food systems for nutrition in Africa: a political economy analysis of food policy in South Africa and Ghana

**DOI:** 10.1017/S1368980024001356

**Published:** 2024-11-28

**Authors:** Anne Marie Thow, David Neves, Robert Aidoo, Linda Nana Esi Aduku, Busiso Moyo, Charles Apprey, Florian Kroll, Reginald Annan

**Affiliations:** 1 Menzies Centre for Health Policy and Economics, Sydney School of Public Health, The University of Sydney, Sydney, NSW 2006, Australia; 2 Independent Researcher, Bellville, South Africa; 3 Kwame Nkrumah University of Science and Technology, Kumasi, Ghana; 4 School of Public Health, University of the Western Cape, Cape Town, South Africa; 5 Institute for Poverty, Land and Agrarian Studies, University of the Western Cape, Cape Town, South Africa

**Keywords:** Food system, Ghana, South Africa, Double-burden, Policy, Nutrition, Agriculture

## Abstract

**Objective:**

To examine underlying political economy factors that enable or impede the integration of nutrition considerations into food system governance.

**Design:**

Comparative political economy analysis of data collected through (1) value chain analyses of selected healthy and unhealthy commodities and (2) food system policy analyses, using a theoretical framework focused on power, politics, interests and ideas.

**Setting:**

Ghana and South Africa.

**Participants:**

Value chain actors relevant to healthy and unhealthy foods (Ghana *n* 121; South Africa *n* 72) and policy stakeholders from government (Health, Agriculture, Trade and Industry, Finance), academia, civil society, development partners, Civil Society Organization (CSO) and private sector (Ghana *n* 28; South Africa *n* 48).

**Results:**

Nutrition was a stated policy priority in both countries; however, policy responsibility was located within the health sector, with limited integration of nutrition into food system sectors (including Agriculture, Trade and Industry). Contributing factors included a conceptions of policy responsibilities for nutrition and food systems, dominant ideas and narratives regarding the economic role of the food industry and the purpose of food system policy, the influence of large food industry actors, and limited institutional structures for cross-sectoral engagement and coordination.

**Conclusions:**

Integrating nutrition into multi-sectoral food policy to achieve multiple food system policy goals will require strategic action across jurisdictions and regional levels. Opportunities included increasing investment in healthy traditional foods, strengthening urban/rural linkages and informal food systems, and strengthening institutional structures for policy coherence and coordination related to nutrition.

The nutrition transition, in which traditional diets are replaced by increasingly processed ‘modern’ diets, is well underway in sub-Saharan Africa, and the region is now characterised by a double burden of malnutrition^(1)^. Undernutrition persists, albeit with some decline, and more than a third of all deaths in sub-Saharan Africa are now due to diet-related non-communicable diseases (NCD), which include diabetes, CVD and various cancers^(1,2)^. Rates of NCD are set to continue to increase; intakes of processed foods, often high in salt, fat and sugar, have risen in most sub-Saharan Africa countries, and the consumption of traditional grains, pulses, fresh fruits and vegetables is declining^(3,4)^. A key contributor to the double burden of malnutrition is the food environment in which consumers make decisions about food purchase and consumption and form dietary habits – shaped by marketing, price and retail practices^(5)^. ‘Food environments’ refer to the surroundings and conditions in which people make decisions about the food they acquire and consume, encompassing availability, accessibility, desirability, convenience, marketing and product characteristics^(6)^.

The United Nations Food Systems Summit in 2021 drew attention to the important role of governance in shaping food systems outcomes across multiple dimensions, including social, environmental, nutritional and economic. Food system governance entails public and private sector actors, as well as civil society actors, who shape decision-making and activities related to the production, distribution and consumption of food; it thus includes the dynamic between the formal functions of government as well as markets, traditions, networks, interests and power^(7)^.

From a public health nutrition perspective, strengthening food system governance such that nutrition is considered, is critical for improving nutritional outcomes^(8)^. In order to temper a full epidemic of diet-related NCD, governments must take policy action to address obesogenic food environments and promote healthier food environments through food systems transformation^(9)^. However, although ministries of health tend to hold policy responsibility for nutrition, policies that influence the price, accessibility and acceptability of healthier (compared with less healthy) foods are largely within the mandate of ministries of finance, trade, industry/investment and agriculture/fisheries, which effectively govern the food system, rather than ministries of health^(9,10)^. The location of food system policy in predominantly in the economic sector and the complex interplay of the formal and informal food sectors in shaping the food environment create challenges for policy-makers seeking to improve diets and health, both globally and within the region^(8,11,12)^. Nutrition is often a stated policy priority, but its integration into food system policies remains limited^(13,14)^. Food is a major economic sector and, across the supply chain, a significant contributor to Gross Domestic Product (GDP) in low- and middle-income countries^(15)^. Previous research in Zambia, Uganda, Ghana and South Africa has shown that operationalising multi-sectoral nutrition policy to ensure that food systems deliver adequate nutrition is currently hampered by policy incoherence, a lack of leadership, a narrow focus on undernutrition and the pre-eminence of economic considerations^(14–16)^. One contributor to this has been the influence of private and corporate interests on food systems governance^(17)^. For example, private capital interests have tended to be prioritised over collective concerns in agriculture in sub-Saharan Africa countries, including in relation to land tenure and environmental sustainability^(18)^, leading to wider calls for food governance reform. As such, there is growing recognition that the political economy of diet-related NCD prevention must be considered due to the political influence of food industry interests on food-related policy^(19)^. However, specific analysis of these dynamics in the region has been limited.

Understanding food systems governance can help public health nutritionists to engage strategically with key stakeholders, to increase consideration of nutrition and support improved food systems outcomes. In this paper, we selected South Africa and Ghana as case studies to examine the politico-economic dynamics of food systems governance. These two countries are relatively advanced in the nutrition transition within the African continent and have significant NCD epidemics. Our overall aim is to better understand the opportunities for elevating consideration of nutrition within food system policy-making, to inform policy engagement in South Africa, Ghana and regionally.

## Methods

The aim of this study was to analyse the political economy of food systems and food system governance in South Africa and Ghana. Our primary research questions were *What are the politico-economy dynamics that influence consideration of nutrition in food system governance?* and *What are the opportunities and challenges for prioritising nutrition within food policy?*

### Study design

We conducted a comparative political economy analysis of national food systems, with a focus on governance, in two phases. The research teams in each country drew on value chain analysis approaches and policy analysis approaches to collect and analyse data (described in detail below). The case study countries were selected as being indicative of a relatively established nutrition transition in the region, but with very different food systems that are likely to provide insights relevant to the diversity evident in the region (Box 1). Here, we present a secondary integrated political economy analysis conducted collaboratively by the research teams from both countries, focused on the interface between food systems and food policy, to identify key contextual factors and power dynamics influencing food policy in both countries, in order to improve understanding of how public health actors could increase consideration of nutrition within food system policy.

### Theoretical framework

We drew on a new institutional approach to political economy analysis to inform the study design and analysis^(20,21)^. Political economy is concerned with power and resource dynamics in a given context and their implications for outcomes; in this case, for human health and nutrition. Political economy analysis examines the interests, incentives and institutions that lie beneath formal structures and enable or constrain change^(20,21)^. Drawing on these theoretical perspectives, and with reference to our study aims, we focused on interests, power and ideas in policy-making related to nutrition and the food system. In particular, we considered underlying discourses, ideas and paradigms regarding the food sector in the context of institutional structures and mandates and how these relate to actor interests and the exercise of power, with a view to identifying challenges and opportunities to increase consideration of nutrition in food system policy^(22)^.

### Data collection

There were two sources of data that supported this analysis, from value chain analysis and policy analysis studies conducted in each country during 2018 and 2019.

Value chain analyses focused on key commodities relevant for the nutrition transition were conducted in Ghana and South Africa. These were designed based on consumer-oriented supply chain analysis^(23)^ and included consideration of actor interests and factors influencing decision-making throughout the supply chain. In each study site, we traced supply chains from the point of consumer interface (i.e. retail) ‘backwards’ or upstream to producers. The focal products were selected healthy foods (leafy greens and fish, as well as cowpea in Ghana), chicken and unhealthy (obesogenic) foods (viz. sugar-sweetened beverages and sweet confectionary/biscuits), to identify linkages, industry dynamics, governance issues, livelihoods/employment considerations, intersections between formal and informal food systems, and constraints. In Ghana, the team conducted interviews based on semi-structured instrument combined with some key informant interviews with retailers (*n* 70), wholesalers (*n* 21) and primary producers (*n* 30) in three out of the sixteen regions of Ghana, including greater Accra, the national capital. In South Africa, fifty enterprise visits and twenty-two in-depth semi-structured interviews were conducted with prepared food and grocery retail enterprises, wholesalers and food manufacturers. They encompassed various sectors (formal and informal) and scales (from informal food vendors to large corporate-owned supermarkets) and were conducted in urban Cape Town and the rural Eastern Cape Province (Mount Frere).

Second, we conducted food policy analysis in each country focused on current food system governance, actors and power. In-depth semi-structured interviews were conducted with key informants in Ghana (*n* 28) and South Africa (*n* 48). We identified potential interviewees from key regulatory and decision-making bodies, as well as from food-related private sector and civil society organisations, involved in national- and local-level food system policy and governance and used snowball sampling to identify further participants. Recruitment was through formal letters of invitation to heads of agencies, followed up with emails/phone calls, to identify appropriate participants. Interviews were tailored to the expertise of the participant and designed to understand current policy priorities, agendas and processes related to food and nutrition, and institutional structures, actor interests, exercise of power, and influences on decisions, with reference to our study frameworks. The interview questions were designed to elicit underlying discourses, ideas and paradigms relevant to food systems policy-making. We also asked participants about opportunities and points of leverage for policy change to strengthen food policy decisions in terms of consideration of nutrition (both in general and regarding the specific commodities of interest: fresh fruit and vegetables, cowpeas, fish, poultry, high sugar food and beverages).

### Analysis

The data for this study were collected as part of the Researching the Obesogenic Food Environment (ROFE) project^(24)^. For this analysis, the raw data were analysed by the study leads from South Africa and Ghana, using qualitative approaches with reference to the study aim, research questions and theoretical frameworks. The political economy analysis presented in this paper relied on secondary analysis of the data from the value chain analyses and policy analyses to synthesise findings related to the political economy of food system governance in both countries. The author team first collaboratively developed a matrix for collation of relevant data based on the study frameworks, which included context, power, political and policy dynamics, actor interests, ideas and frames, gender and influences on the consumer food environment, relevant to value chain analysis and policy analysis in each study country. The study team collaboratively analysed the matrix to identify key findings relevant to understanding the political economy of food systems and their governance, and similarities and differences between South Africa and Ghana. Following this, the lead author further analysed the synthesised data and explicated the key emergent themes of actor interests with respect to food system policy priorities, how power is exercised in food system policy, ideas about the role of the food system (including with respect to nutrition) and sectoral dynamics (specifically, the role of the Ministry of Health). The study team reviewed and refined these findings iteratively to develop the final study results.

Box 1. Characteristics of case study countries

South Africa is a middle-income country with high levels of inequality and poverty. The food system is highly deregulated and industrialised, dominated by large domestic and multinational food corporations, and is integrated into the global economy through both trade and foreign investment (inwards and outwards)^(17,25)^. Formal agri-food supply chains and supermarkets predominate^(26)^. South Africa’s food system makes a significant contribution to the national economy; in 2016, the food retail industry alone contributed 9 % to overall GDP^(26)^. However, governance challenges have been repeatedly identified, including policy incoherence across the sectors governing the food system with respect to meeting economic, food security and nutrition objectives^(15)^ and with inadequate coordination with respect to food security^(26)^. The South African economy and food system are characterised by extreme inequality in terms of health outcomes; one in four South African children are stunted, 68 % of women and 31 % of men are overweight or obese^(27)^, and about 20 % of South African households experience food insecurity^(28)^. To date, the state welfare payments (‘social grants’) have been a primary food security mechanism; but these are insufficient without broader food system strengthening^(29)^.

In contrast to South Africa, Ghana’s food system is at an earlier stage of industrialisation. The contribution of the agricultural sector to the economy remains significant, but local food production (e.g. for staple food crops such as roots, tubers and vegetables) is inconsistent and the priority for the agriculture sector is increasing production through modernisation^(30)^. The food system is ‘mixed’, with traditional retailing (e.g. open markets) prevalent across the rural and peri-urban areas in the country and modern retailing (e.g. supermarkets and small shops) in the bigger cities. The processed food sector is dominated by imports, which comprise about three-quarters of products, although domestically processed foods are increasingly available in large urban centres^(31)^. Traditional diets are still common, particularly among older people, and there is strong cultural resonance for traditional staple foods, including cassava, maize, plantain and yams^(31,32)^. Ghana has made progress in reducing the prevalence of undernutrition; however, the country is experiencing a double malnutrition burden. The prevalence of stunting among children remains about 20 %, and 42 % of women of reproductive age are Fe-deficient^(33)^. At the same time, 16 % of men and 40 % of women are overweight or obese^(33)^, and the prevalence of diet-related NCD are rising, associated with urbanisation and increasing affluence which can increase purchasing of ultra-processed foods^(34)^.

These two countries have similarities and differences in their food systems. South Africa is significantly further along in both food system industrialisation and the nutrition transition, but Ghana is also one of the countries within the region rapidly undergoing the nutrition transition. Regional expansion of South Africa’s food industry has entailed the entry of its multinational supermarket retailers across the continent including into Ghana. Both countries also face persistent economic and development challenges, including ‘jobless growth’^(35)^, and the food and agriculture sector has been identified as an important economic sector^(36)^. In both countries, as in the region more broadly, historical colonial (and in South Africa, apartheid) legacies have also had a major influence on the ongoing development of food systems and food system policy^(37)^.

## Results

### Nutrition and food system policy priorities

In both countries, the policy analysis indicated that addressing malnutrition and food insecurity was a stated national policy priority and with leadership from the national development agency (National Development Planning Commission in Ghana and The National Planning Commission in South Africa). This enabled a whole-of-government approach to (under)nutrition and food security, with national coordination for multi-sectoral action. It was evident that food security in particular had been integrated into sectoral mandates related to agriculture and welfare (Social Development) and nutrition into health sector mandates.

With reference to the nutrition transition, however, it was evident that the policy priority for (mal)nutrition at the central government level in both countries did not extend to diet-related NCD. It was within the health sector (i.e. the Ministry/Department of Health) that there were specific policies to address both undernutrition and NCD. Overall, resource allocation and technical expertise related to nutrition were still focused on undernutrition and nutrition-specific interventions. While the Ministry/Department of Health in both countries formally recognised the importance of healthy food supply to support nutrition objectives, they had no formal remit for policy instruments relevant to food governance.

Food policy decisions were within the purview of Agriculture, Industry and Trade sectors. Interviews with food system actors and food policy actors in both countries indicated little active consideration of nutrition by food system actors or policy. In both countries, cross-sectoral coordination to integrate nutrition into sectoral mandates and to manage conflicts of interest remained challenging. Policy respondents across sectors recognised that unhealthy diets were associated with poor health outcomes but emphasised individual/consumer-oriented reasons for citizens eating unhealthy diets, such as a lack of knowledge. As a result, they located responsibility for addressing nutrition with the Ministry/Department of Health and with individuals. Interviewees within both Ministry/Departments of Health in turn reported that engaging across sectors to promote healthier food environments was challenging. This was in part due to the ‘responsibility’ of the health sector for nutrition, which created challenges for action in food system sectors, which had clear economic and livelihood mandates related to trade, industry, finance and production. One element of the broader context of this effective separation of food policy mandates from nutrition policy was very different framing of engagement with food industry actors by policy actors. In the economic food policy sectors, (formal) industry was one of the primary stakeholders in policy, and there was significant attention to their concerns and interests. In contrast, in the health sector with respect to nutrition, there was limited engagement with industry actors (other than with respect to food safety, specifically). Interviewees from the health sector raised concerns about potential conflicts of interest regarding nutrition and economic objectives related to the food system, particularly with respect to regulation of the food environment (e.g. labelling and marketing restrictions).

In interviews for this study, policy-makers within economic sectors often expressed interest in identifying new approaches to achieving objectives – both economic and broader social goals. For example, in South Africa, there was growing recognition that food system outcomes were contributing to ongoing inequalities and not fully achieving social, health or economic goals. In Ghana, there was a sense that current approaches to economic development were achieving limited benefits for the food sector and that human development was being hampered by persistent undernutrition. These factors suggested opportunities and goodwill in both study countries for considering new approaches to food system governance that would better support integration of nutrition as a priority within policy sectors that govern the food system.

The political economy analysis presented here, which brings together the policy analyses and value chain analyses conducted, was thus focused on understanding the disconnect between the evident priority for improving nutrition at the national level and within the health sector, and the limited translation of this into food system policy and action. By examining the interests, incentives and institutions that lie beneath formal structures and enable or constrain change, we sought to shed light on the persistence of the policy status quo that is resulting in poor nutrition (and other) outcomes.

### Interests of food system actors

In both study sites, we examined the interests of food system actors and food policy actors. The primary considerations of food industry actors were, unsurprisingly, economic in nature, but they varied by type of industry actor. In Ghana, the agricultural sector was dominated by resource-poor smallholder farmers, who are the main suppliers of local food stuffs to traditional open markets, and also have a significant subsistence concern. In contrast, South Africa is not an agricultural-based economy, and small producers made a fairly small contribution to the food supply (agriculture represents less than 3 per cent of the country’s GDP compared with Ghana’s 17 per cent). In both countries, the evident interests of small farmers were reported as livelihoods and subsistence, while larger farmers sought to attain and increase access to domestic value chains through enhancing efficiencies and production volumes (agricultural ‘modernisation’, and particularly integration into formal value chains). In Ghana, in particular, improving market access and wastage were identified as significant interests by food system actors, although small producers had limited interest in accessing international value chains through trade. In contrast, agricultural production in South Africa is dominated by large commercial agriculture entities, often concerned with liberalising international trade. It was notable that the dominance of large-scale commercial farming in South Africa has deepened with post-apartheid liberalisation and agricultural market deregulation, as well the preference of large manufacturers and retailers for ‘stable’ supply (see below on trends evident in Ghana).

The profit and long-term economic concerns of large agribusiness and food industry were expressed primarily in an emphasis on sector growth and access to global value chains, and, in the case of South Africa, regional expansion. In South Africa, large national and multinational food processors and retailers had strong policy interests in liberalising trade and investment policy, and domestic deregulation. In contrast, these large industry actors in Ghana were primarily wholesalers and retailers (large supermarket chains are fewer in number, and they deal mostly in imported commodities), and their policy interests reflected the less organised production sector, in particular, strengthening domestic supply chains and access to imports to offset fluctuations and limitations in domestic supply.

Priorities articulated by food policy actors across sectors governing food systems (namely, Agriculture, Trade and Industry sectors) in both countries primarily focused on economic considerations, including trade, employment and economic growth. Policy-makers in food system sectors referenced both industry interests, as described above, and national economic priorities (namely, employment). Policy priorities were oriented to the interests of larger and formal industry actors, in particular, priorities related to active investment in and incentives for increasing and facilitating food trade, which focused on value-adding and connecting domestic industry actors with international value chains. Formalisation of the food system was also a priority, particularly integration of small-scale farmers into the formal (industrial) food system. The latter was notable in the nature and focus of South Africa’s support for black (indigenous) entrepreneurs, which included public–private partnerships with major food industry actors. In contrast, although modernisation of agriculture was a policy priority in Ghana, the small-scale nature of agriculture in Ghana created challenges, especially the lack the infrastructure, knowledge and capacity among farmers. Consumer interests were also clearly evident in food system policy. In both countries, we found that governments were concerned with food safety as well as stability in food prices.

### Power in food system governance

In both countries, we identified alignment between the interests of large agri-food system actors in the value chains and the industrial (and agriculture) policy focus on value addition, processing and trade, although this was tempered in Ghana by the significance of small-scale farmers and limited number of large agri-food industry actors. The interview data indicated two key avenues through which industry actors exercised power in the policy space. First, interviewees in both countries indicated that the alignment of industry interests with dominant government policy priorities for economic growth and employment meant that their specific policy-relevant interests were very influential. In Ghana, for example, wholesalers and importers are well resourced and powerful in the food system and able to provide support to politicians. These actors have effectively lobbied to liberalise trade and limit restrictions on imports, even though high volumes of imports have tended to stifle domestic production capacity (i.e. in the domestic broiler and rice industries). Large industry has also tapped into concerns about employment to curtail regulation in both countries. For example, in South Africa, poultry producers lobbied against the relaxation of import tariffs on imported frozen chicken. However, it was also evident that their influence reflected the disproportionate visibility and policy salience of formal employment and formal sector revenue generation, compared with the smaller-scale informal sectors.

Second, the interviews with value chain and policy actors indicated that knowledge was a source of power: industry positioned themselves effectively as knowledge holders or technical experts. In South Africa, large industry was effectively positioned as a ‘knowledge holder’ to inform policy decisions and also as experts in food system development, for example, through public–private partnerships in which large industry mentored smallholder farmers and small manufacturers. In Ghana, a lack of knowledge among farmers and other value chain actors was articulated as a reason for food system ‘failure’, for example, the decline of the domestic chicken industry. This narrative functioned to reinforce arguments for modernisation and more ‘developed’ industry actors as essential to achieving food system goals in Ghana.

Formal responsibility for food policy was quite separate from nutrition policy, and within food policy forums, industry tended to be influential. In South Africa, there were evident disconnections between jurisdictions of government (viz. national, provincial and local), with food largely viewed through lens of ‘food safety’, which is vested in local government as ‘environment health’. Nutrition was not within their purview. Similarly, in Ghana, food safety was strongly regulated by the Food and Drugs Authority. In both countries, there were complex dynamics across jurisdictions within food system sectors, in terms of National Agriculture (with subnational programmes), National Trade & Industry (with subnational programmes to foster export-oriented Small and Medium Enterprises), and Provincial and Municipal Urban Planning. Within all of these sectors, there were multiple forums for consultation and input by the private sector. In both countries, planners and developers were hugely influential on urban planning policy and policy related to food retail, with a strong focus on modernisation and the formal sector. The urban–rural interface within the food systems was largely ignored, and municipal planning processes were rarely linked to agriculture policy or to nutrition policy.

In both South Africa and Ghana, there was an indication of growing civil society advocacy regarding food systems and the right to food. However, the influence of the civil society voices in both countries appeared limited (effectively fragmented) because of both the multiple spaces for decision-making described above and because of the multi-dimensionality of the ‘food policy’ issues being advocated for. In effect, advocacy regarding environmental sustainability, livelihoods, inequality, food security, nutrition and (in South Africa) the right to food were separate advocacy issues. This meant that even where consultation structures exist for policy-making, civil society voices were often marginalised. In addition, there was not a strong constituency related to prevention of either chronic undernutrition or diet-related NCD, because the deleterious effects are invariably long term, have systemic causes and tend to be individualised.

### Conceptions of the food sector

We identified three dominant ideas or narratives about the food system and food system governance in both countries that helped us understand the sway of large industry over policy and the limited consideration of nutrition. First, evident in both countries was a shared perception among food industry and food system policy actors that the role of the food sector in relation to nutrition was simply to produce sufficient food and that issues of nutritional quality – apart from technical action on reformulation and fortification – were a marginal or side concern for the food industry. This was reflected in an overriding policy paradigm of sufficiency in food policy evident in both countries. Although industry actors in South Africa recognised diet-related health concerns, it was clear that ‘nutrition’ was seen as a concern for a niche market, with potential for sector growth arising from growing middle-class concerns about diets and health. In Ghana, there was more attention to undernutrition and sufficiency from the industry side, with little concern regarding diet-related NCD. However, value chain actors often did not perceive that they had any role in judging the nutritional quality or influencing the supply of ‘healthy’ foods, with food framed and managed as a commodity. An underlying narrative was evident in both countries that the food industry is simply responding to consumer demands in a competitive environment and that it is up to consumers to choose healthier options.

Second, in both study sites, we found that nutritional quality and by extension diet-related NCD were simply ‘externalities’ and that there was structurally no incentive for food system actors to consider these factors. There were no evident incentives – policy or otherwise – to ‘internalise’ the costs of nutritionally suboptimal foods (e.g. ultra-processed foods, high-energy–low-nutrient foods, etc). In fact, in value chain terms, minimally processed food had significant disadvantages for food manufacturers and retailers due to limited scope for value-add, challenges with product differentiation and branding, their perishability and presentation often in non-standard units. In contrast, food safety issues were strongly internalised. For example, in South Africa, major corporate efforts on food safety were evident because food-borne illness events exacted reputational costs, which translate into potentially large financial losses.

Third, there was a shared belief between food industry and food system policy actors that (formal, large) industry is the appropriate – indeed predominant – pathway to economic growth. Industry effectively positioned itself – and was viewed by economic policy-makers – as important to the economy and society. In both countries, large industry was seen as imperative for economic efficiencies and scale of production required to meet growing domestic demand. It was also linked to urbanisation and need for effective domestic distribution, which large retailers were seen by economic sector interviewees as being able to achieve. Informality was seen as undesirable for a ‘modern’ economy – but at the same time somehow necessary in a ‘transitional’ context. There was thus an absence of policy priorities for, and policy engagement with, the informal sector in both countries other than a general push for formalisation (in other words, an apparent implicit assumption that informality will eventually be eclipsed by development). The ‘informal’ food industry in both countries was characterised by the lack of a coordinated approach, limited recording keeping, organisation and lobbying. However, in both countries, the informal sector constituted a significant element of the food system and retailed both minimally processed fresh foods and ultra-processed products. There was also significant interplay and flows of products from the ‘formal’ to the ‘informal’ food sector, particularly in South Africa.

A significant difference between the two study countries was the prevalent discourse regarding indigenous traditional (largely healthy) foods in Ghana, such as cocoyam leaves (*kontomire*), other indigenous leafy greens and cowpeas. This discourse was not evident at the policy level in South Africa, where the historical disruption of indigenous foods and food culture sees many contemporary ‘traditional’ dishes constituted from ingredients (chicken, maize, sugar, etc) produced by the industrial food system. In Ghana, the persistence of minimally processed traditional foods and cuisine also meant that there was a clear identity regarding the primary producers of these foods, despite the shift towards imported food items and highly processed foods.

Another notable difference was the health promotion levy in place in South Africa, which is a tax on sugar-sweetened beverages. Interviewees noted the successful adoption of the tax as an indication of public health nutrition interests being recognised as important in the economic policy space. However, the reduction of the proposed tax rate by half in the final regulation was the result of intense lobbying activity by the sugar industry, which also mobilised support from the Congress of South African Trade Unions (COSATU) and appealed to economic interests through potential job losses.

## Discussion

This study explored the political economy of food systems policy to better understand the limited integration of nutrition into food systems policy across various sectors. In both case study countries, the food system is large, complex and lucrative, marked by powerful actors and vested interests. The food systems in the two countries have differences, particularly with respect to the level of industrialisation, but it seems as though the current trajectory in Ghana will result in a situation similar to South Africa – with a focus on commercialisation of agriculture and industrialisation of the food supply – which is clearly generating adverse nutritional impacts. By selecting two study countries that are both among the most industrialised and furthest along in the nutrition transition in the region, this study offers insights that can inform earlier intervention in other countries in the region, in particular, the potential to strengthen consideration of nutrition in food system policy through: shaping narratives regarding nutrition and its location within policy-making, strengthening institutional structures for cross-sectoral engagement, reorientation of food system policy investment and attention towards healthier foods and revisiting the role of regional agencies in nutrition as well as food security.

This analysis revealed the pervasive discourses sustaining a governance system in which the mandates for food systems policy and nutrition are effectively separated. This division is further strengthened by the discursive appeal to job creation and economic growth as a political priority, while the nutritional wellbeing of people was implicitly of secondary importance and the outsized influence of industry on economic (food system) policy. This reflects previous findings regarding the limitations of current ‘productionist’ agricultural models that exist across the African region in achieving multiple policy objectives^(18,38)^ and regarding food industry discourses^(39)^. It also reflects global productivist paradigms narrowly focused on food sufficiency and industrialisation^(18,40,41)^. Although we found some interest in alternative approaches to food systems policy, especially in Ghana, this did not seem to be translating into concrete changes in the policy approach. This finding is in line with other food system research, and there appeared to be limited awarness among policy-makers about alternate approaches to food systems that could achieve multiple policy objectives^(42)^. While minimally processed traditional diets remain culturally resonant, there is potential for investment in traditional foods and their value chains to meet demand and contribute to innovation for economic diversification and development^(43)^. However, the producers of fruit, vegetables and traditional staples were largely overlooked by policy and wielded little power within the supply chain, despite previous research suggesting market opportunities for healthy traditional food in both South Africa^(44)^ and Ghana^(32,45)^. The advent of COVID in the year following this study has raised the profile of nutrition and health (with obesity a risk factor for poor COVID-related outcomes) and has also drawn attention to the fragility of food systems globally. There is a potential policy window for increasing support for the informal food sector, with growing recognition of the strengths of this sector and its relatively high level of resilience^(46)^.

The findings of this study point to the need to reconceptualise ‘nutrition’ policy outside of the health sector. In the two study countries, powerful actors generated negative (health) externalities with little evident discourse on the necessity of policy intervention to regulate the food industry. By taking a broad food systems and political economy approach, this study was able to identify that the ‘location’ of nutrition policy within the health sector may prevent concerns about nutritional externalities from influencing industrial, trade or economic development policy. This underscores the importance of future analyses of food systems for nutrition focusing on reorienting economic systems and economic priorities towards sustainability (on multiple fronts) and equality^(18)^. In particular, the policy push towards commercialisation and modernisation in Ghana, with limited explicit consideration of nutritional outcomes of the food system, suggest that attention should be given to framing ideas related to nutrition and food systems. However, efforts to broaden nutrition mandates outside of the health sector must engage with the different nature of engagement with industry in food system policy-making. Core to health sector policy recommendations – for example, from the WHO – are calls to reduce the influence of vested interests in nutrition policy-making^(47)^. Our study indicated that the strong narratives regarding the centrality of industry to growth and development in the economic (food policy) sectors mean that the nature of vested interests for different sectors must be examined and defined, as a basis for building meaningful cross-sectoral policy coherence.

This study also identified an opportunity for increased policy attention in the region to urban development, with respect to both urban–rural linkages and the informal food system. National, provincial and district/municipal food plans that address environmental, nutritional and economic aspects of food systems in an integrated way have been proposed as a strategy for creating stronger rural/peri-urban/urban linkages^(48)^. Many of the potential policy levers to influence food environments are located at the local government level in South Africa and Ghana, including spatial planning instruments, by-laws, environmental impact assessments and integrated development plans^(49)^. However, strengthening informal food systems in urban areas will require shifts in framing regarding the safety and marginality of these food systems, as well as coordinated policy support^(50)^. In South Africa, there are few explicit food mandates at the municipal level (apart from food safety), and decisions are often driven by economic concerns which prioritise property values and the attractiveness of cities for investment over the livelihood and nutrition needs of the poor. To date, there has been little engagement by the public sector in urban development in Ghana, for example, in development of malls in Accra^(51)^.

The main strength of this study was the integration of value chain and policy analysis approaches, drawing on multidisciplinary research expertise from agriculture, political science, economics and human nutrition. This enabled us to examine power dynamics and policy influence in detail. However, drawing on multidisciplinary approaches across two different country contexts also made it challenging to integrate and compare findings across studies and study sites. In this secondary analysis, we were limited in our ability to conduct an in-depth analysis of the large volume of data, which were first analysed via disciplinary approaches for each country; as such, our analysis is necessarily high level and focused on major policy dynamics. This invariably overlooks nuances within study sites.

### Conclusion

Nutrition is still seen as a health sector issue across the African region, but averting an escalating double burden of malnutrition and (unaffordable) NCD epidemic will require integration of nutritional considerations into food system governance. Analysing where power lies and the paradigms and ideas that underpin current food system policy can provide insights to inform broader health sector engagement with food policy sectors. Understanding the governance dynamics of these other sectors offers potential for mutual benefits arising from effective action on the current inequalities and negative externalities within the food system. This political economy analysis identified specific opportunities to increase consideration of nutrition in food system policy in South African and Ghana, with implications for other countries in the region. These included increasing investment in healthy traditional foods, strengthening urban–rural linkages and informal food systems and strengthening institutional structures for policy coherence and coordination related to nutrition.
